# New aspects of *Saccharomyces cerevisiae* as a novel carrier for berberine

**DOI:** 10.1186/2008-2231-21-73

**Published:** 2013-12-20

**Authors:** Roshanak Salari, BiBi Sedigheh Fazly Bazzaz, Omid Rajabi, Zahra Khashyarmanesh

**Affiliations:** 1 Department of Drug and Food Control, School of Pharmacy, Mashhad University of Medical Sciences, Mashhad, Iran; 2 Biotechnology Research Centre, School of Pharmacy, Mashhad University of Medical Sciences, Mashhad, Iran; 3 Targetted Drug Delivery Research Centre, School of Pharmacy, Mashhad University of Medical Sciences, Mashhad, Iran

**Keywords:** Berberine, Encapsulation, *Saccharomyces cerevisiae* yeast cells

## Abstract

**Background:**

Berberine was encapsulated in yeast cells of *Saccharomyces cerevisiae* as novel carriers to be used in different food and drug industries. The microcapsules were characterized by differential scanning calorimetry (DSC), fourier transform infra red spectroscopy (FT-IR) and fluorescence microscopy. The encapsulation factors such as plasmolysis of yeast cells which affects the % encapsulation yield were studied.

**Results:**

Fluorescence microscopy showed the yeast cells became fluorescent after encapsulation process. DSC diagram was representing of new peak for microcapsule which was not the same as berberine and the empty yeast cells peaks, separately. FTIR spectrums of microcapsules and yeast cells were almost the same. The plasmolysed and non plasmolysed microcapsules were loaded with berberine up to about 40.2 ± 0.2% w/w.

**Conclusion:**

Analytical methods proved that berberine was encapsulated in the yeast cells. Fluorescence microscopy and FTIR results showed the entrance of berberine inside the yeasts. DSC diagram indicated the appearance of new peak which is due to the synthesis of new product. Although plasmolysis caused changes in yeast cell structure and properties, it did not enhance berberine loading in the cells. The results confirmed that *Saccharomyces cerevisiae* could be an efficient and safe carrier for active materials.

## Background

Microencapsulation is a process that nowadays developed in many industries. Encapsulation has a lot of advantages. The encapsulated compound can be released in a controlled way in different systems. Besides, it stabilizes the active materials against oxygen or other molecules by wall material as a physical barrier [1]. The stability and release properties of microcapsules are dependent on cell wall properties [2,3]. However, the new or novel coatings are needed due to the cost and legal limits in the food industries [4].

*Saccharomyces cerevisiae* yeast cell can be mentioned as an ideal carrier due to its food-grade and low cost characteristics. Unlike other carriers, it does not depend on active ingredients for its synthesis. We could culture this kind of carriers as much as needed without any excess expenses. Besides, its membrane phospholipids act like liposome structure and have been used for encapsulation of different molecules, hydrophobic and hydrophilic, like resveratrol [5-8].

It possesses the external thick cell wall which composes of a beta glucan network and a small amount of chitin associated with a mannoprotein layer [9]. These cell wall properties make *S. cerevisiae* such a kind of sustained release drug delivery system. The mechanical characteristics of the yeasts structure allow them to load different molecules.

Barberry (*Berberis vulgaris* L. family Berberidaceae) grows in Asia and Europe. The plant is famous worldwide for its medicinal properties [10]. Berberine is the most significant alkaloid of this plant which is responsible for its beneficial effects. Berberine is mostly accumulated in the root. Berberine as an isoquinoline alkaloid is a member of protoberberines class [11]. Berberine shows a lot of pharmacological effects [12].

Berberine shows antiplatelet effects and it is also used in treatment of congestive heart failure (CHF) [13-16]. Extracts are used to cure various inflammatory diseases such as lumbago and rheumatism and to reduce fever [17]. The reports represented immunosuppressive effect of berberine in the tubulointerstitial nephritis model. Peng et al. showed the antianxiety properties of berberine [11]. Berberine can be mentioned as an expectorant due to its ability to increase mucin release [18].

Berberine has been used to treat infectious diarrhea and gastroenteritis in China in the past [19]. Berberine is defined as an effective drug to cure acute diarrhea which is due to *Escherichia coli* or *Vibrio cholerae*[20,21]. Berberine shows significant antibacterial and antifungal activity against broad spectrum of microorganisms [22-28].

Berberine’s poor water solubility and susceptibility to environmental conditions prevent its application in many industries. The goal of this study was to evaluate the new aspect of yeast cells of *S. cerevisiae* as an encapsulation carrier for berberine. Moreover, the berberine microcapsules were studied by different analytical methods such as fluorescence microscopy, (DSC) and (FT-IR).

## Methods

### Preparation of plasmolysed yeast cells, non plasmolysed yeast cells and berberine microcapsules

The yeast cells (commercially Bakers *S. cerevisiae)* were cultured in soybean casein digest broth medium (Himedia, India) for 10 hours [29] in shaker incubator (20 rpm) (JTSL 20, Iran). The medium was centrifuged and the yeast cells were washed for three times with deionised water. The final cells were classified in two groups. First group as non plasmolysed cells was directly freeze dried. The second one as plasmolysed cells was plasmolysed in different concentration of sodium chloride solutions. The yeast cells suspensions were provided in three different flasks. Each flask contained 10%, 20%, 30% w/w NaCl solutions, respectively. Then the flasks were stirred at 200 rpm for 72 h. The plasmolysed cells were centrifuged and washed three times with deionised water to remove impurities. At last, they have been freeze-dried. The freeze-dried plasmolysed and non plasmolysed yeast cells were studied as carriers [30].

Two kinds of freeze dried cells (plasmolysed and non plasmolysed) (100 mg) were suspended in two flasks containing 50 ml berberine solutions (500 mM). Berberine hydrochloride was obtained from China (XI AN Rongsheng biotechnology CO., LTD). The flasks were stirred at 200 rpm for 72 h and then centrifuged (6000 rpm, 20 min). The precipitants were washed three times to remove the free berberine. Then the berberine loaded microcapsules were freeze dried.

### Analytical methods for confirmation of berberine encapsulation

Three methods were performed for confirmation of encapsulation process including fluorescence microscopy (color CCTV camera, model No.MV-CP470/G), fourier-transform Infrared Spectroscopy (FT-IR) (model. Spectrum two) and differential scanning calorimetry (DSC) (Mettler Toledo).

Fluorescence microscopy images were obtained from empty freeze-dried yeast cells and the yeast cells which encapsulated berberine. Besides Fluorescence microscopy, DSC and FT-IR studies were carried out to confirm the encapsulation of berberine in yeast cells.

In DSC studies, five types of freeze-dried samples were studied: empty yeast cells, berberine powder,physical mixture of berberine and empty cells, cells loaded with berberine (microcapsules) and plasmolysed empty yeast cells. To prepare a DSC sample, a definite amount of each sample was placed in 50 μl closed aluminium pans. The scan rate was 10°C/min between 0 and 400°C. The IR spectra (KBr disc method) of all the above mentioned samples were also obtained using an FT-IR spectrophotometer [8,10].

To determine the encapsulation yield (% EY), berberine was extracted from two kinds of microcapsules. In extraction process, each sample ( 50 mg ) was suspended in deionised water and ethanol by ratio of 1:4. Then the suspensions were stirred for 48 h in room temperature. Each cell suspension was then centrifuged. Berberine in the supernatant was then quantified by UV/Visible spectroscopy. Then the (% EY) was calculated [28]:

$$\begin{array}{ll} \left(\mathrm{\%EY}\right)= & \mathrm{Mass}\;\mathrm{of}\;\mathrm{encapsulated}\;\mathrm{berberine}\\ & ÷\mathrm{Mass}\;\mathrm{of}\;\mathrm{the}\;\mathrm{resulted}\;\mathrm{microcapsule}\times 100 \end{array}$$

## Results and discussion

### Analytical studies of freeze-dried plasmolysed and non plasmolysed yeast cells

**Figure 1:**
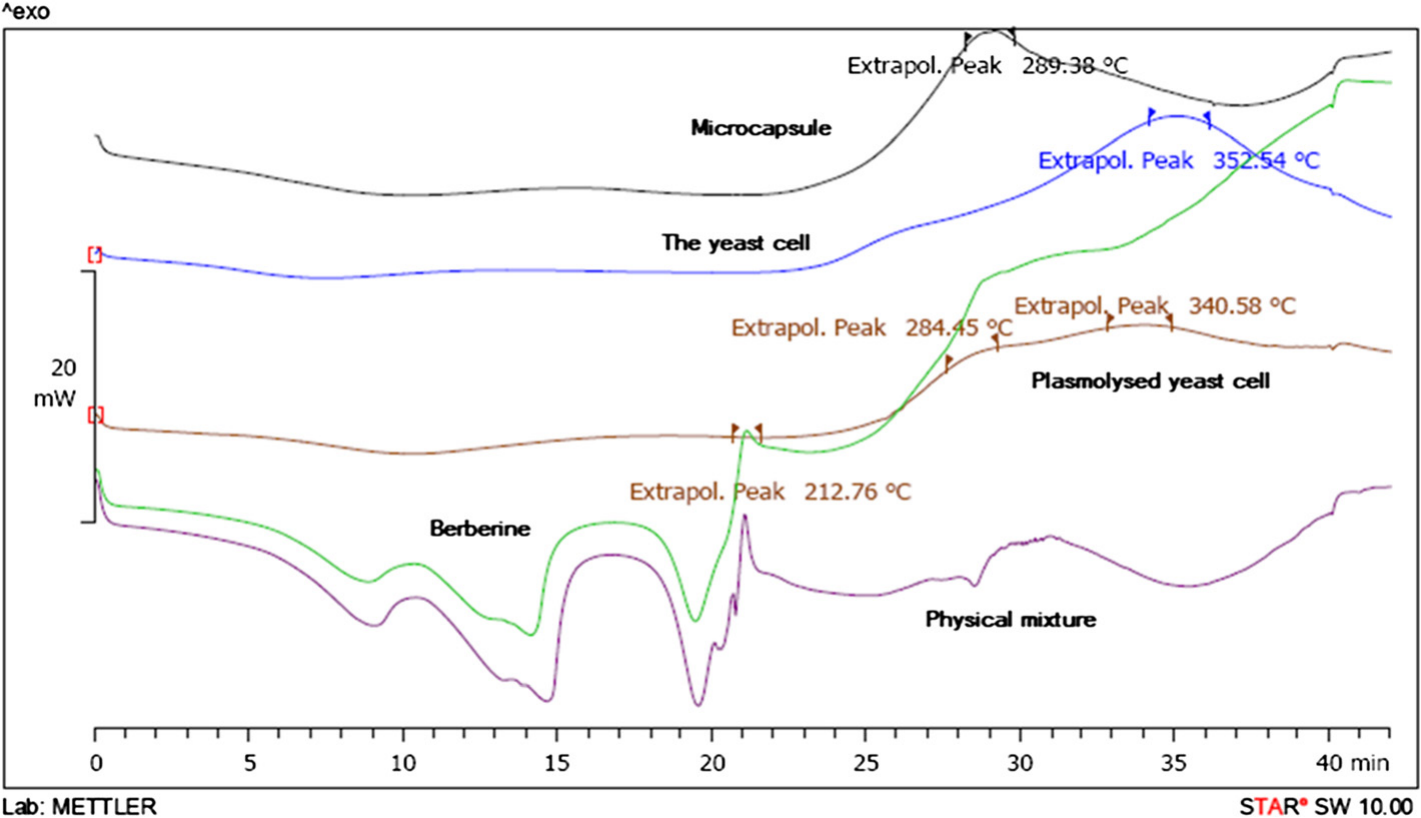
**DSC thermograms of microcapsules, *Saccharomyces cerevisiae *** **yeast cell, berberine, plasmolysed yeast cells and physical mixture of berberine and yeast cells.**

The DSC thermograms are showed in Figure 1. DSC thermograms indicate the heat capacity changes of a product based on temperature or time. Besides, we can prove the formation of a new product by omitting or obtaining of new peaks. The DSC thermogram shows an exothermic peak with maximum occurrence at 352°C for freeze-dried non plasmolysed yeast (Figure 1). This peak is originated from the phase transition temperature (Tm) of yeasts’ phospholipid bilayer and at 352°C the hydrocarbon tail melted [30]. Tm is influenced by membrane structure so the DSC thermogram of the freeze-dried yeasts cells that had been plasmolysed in 20% w/w NaCl solution showed exothermic peaks with maximum occurrence at 284 and 340°C. It confirms that plasmolysis process causes changes in lipid composition of membrane and shows different maximum occurrence in comparison with non plasmolysed yeast cells [31]. Tm also depends on different parameters such as strain of *S. cerevisiae*, so we observed different Tm in different studies.

**Figure 2:**
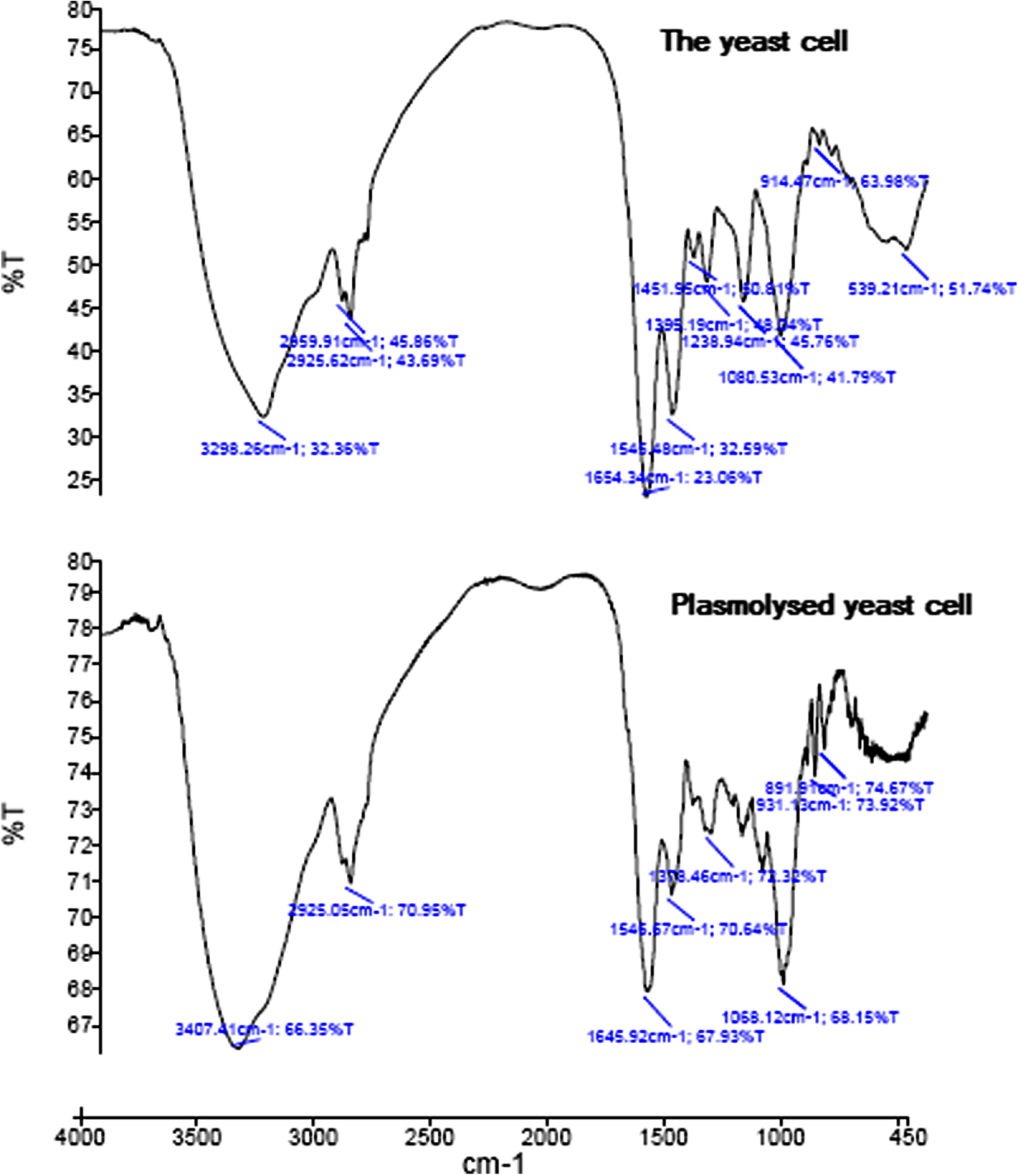
**FTIR spectra of *Saccharomyces cerevisiae *** **yeast cell and plasmolysed yeast cells.**

**Figure 3:**
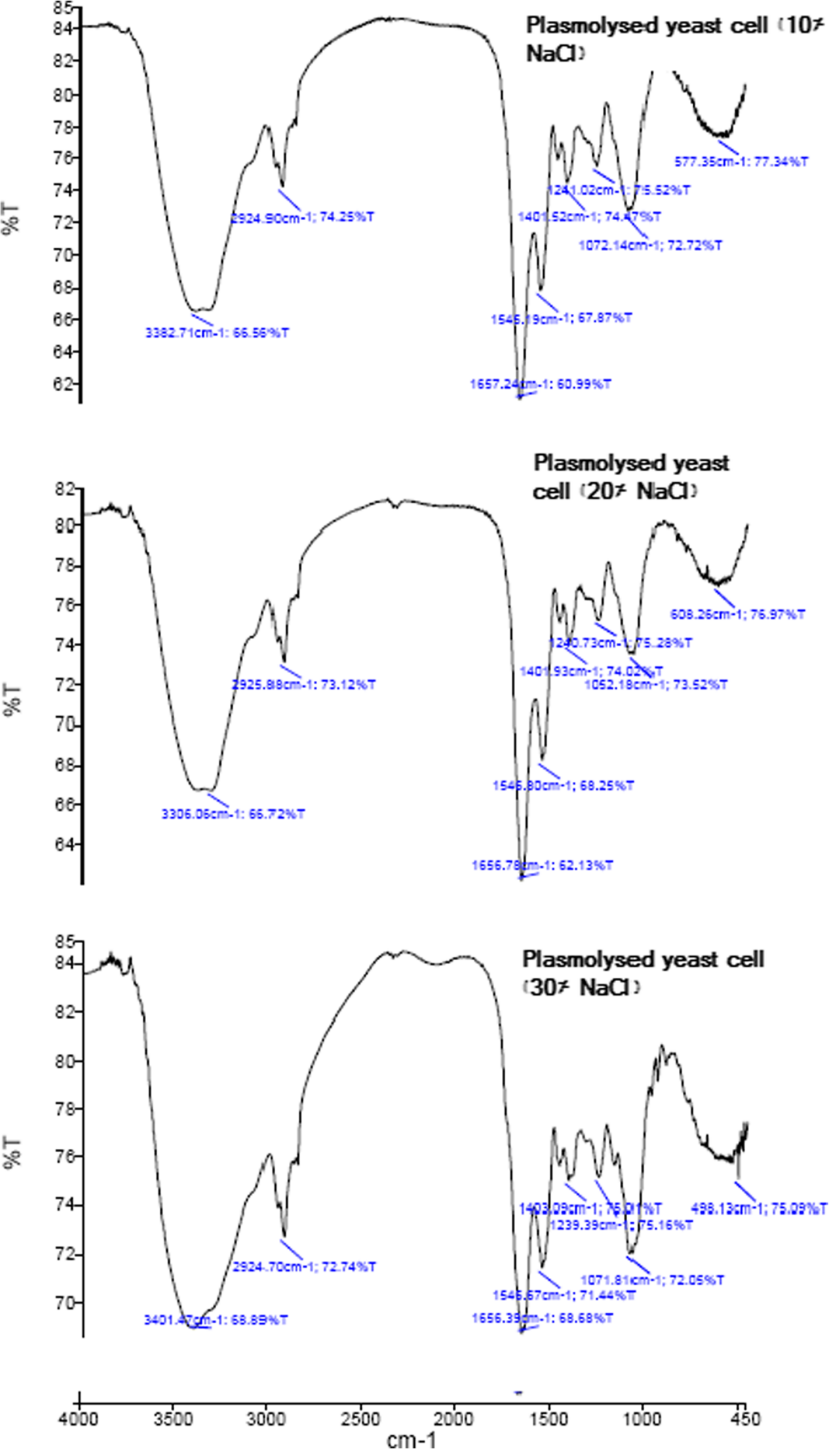
**FTIR spectra of 10%, 20% and 30% plasmolysed *Saccharomyces cerevisiae *** **yeast cells.**

FT-IR was also used to study changes in the cells structure as a result of yeast cell plasmolysis. The IR spectra of both plasmolysed and non plasmolysed freeze-dried yeast cells are shown in Figure 2. The spectra of different plasmolysed yeast cells with 10%, 20% and 30% w/w NaCl solutions (Figure 3) show no significant differences among each other but in comparison with the spectra of non plasmolysed yeast cells (Figure 2), we could see characteristic changes that they represent the effects of plasmolysis on cell wall or membrane compositions such as proteins, carbohydrates, lipids and even nucleic acids.

The contributions of hydroxyl vibrations of carbohydrates and NH vibrations of proteins can be observed in a band at 3750–3000 cm^-1^ in IR spectra [31].

Region 3050–2700 cm^-1^ shows us the information about lipid composition of yeast cells. Plasmolysed yeast cells indicated a minor increase of their absorption intensities due to the changes in the length of membrane lipid chains in comparison with non plasmolysed yeast cells absorption peaks in 2959 and 2925 cm^-1^ (Figure 2) [32]. These two peaks are due to the vibrations of methyl and methylene groups. Plasmolysis effects on cell wall and membrane proteins could be seen in different regions. According to protein degradation by plasmolysis, we faced some changes in peak intensities in the region 1700–1550 cm^-1^. After plasmolysis, some changes happened in the absorption bands at 1659 and 1553 cm^-1^, which referred to protein amide I and amide II and carbon-nitrogen vibrations of yeast cells due to the shift of the degradated proteins to an unfolded state. Degradation of one part of yeast proteins in the cell membrane and the cell wall could be detected in the band region from 1530 to 1385 cm^-1^.

Plasmolysis caused the cell wall polysaccharides degradation which could be detected by changes in the IR region from 1156 to 768 cm^-1^ (Figure 2).

Hypophosphite vibrations are originated from nucleic acid molecules, so we could find the effect of plasmolysis on nucleic acids degradation as the 1240 cm^-1^. Nucleic acid molecules are placed inside the cell so the presence of their IR spectra are indicative of cells which are not completely emptied by plasmolysis (Figure 2).

Finally the results show us that plasmolysis causes disorganisation to the cell plasma membrane and thickness of cell wall but higher concentrations of NaCl solutions showed no additional disruptions in the yeast cell.

Plasmolysis enhances the intracellular space so it improves the capacity of carriers for encapsulation process [33]. Nowadays, yeast cells plasmolysis with NaCl [7] were mainly used for the encapsulation of different active materials.

The results indicated that the %encapsulation yield values were statistically similar (p > 0.05) amongst plasmolysed and non plasmolysed cells which confirms other researches results [30]. The average %encapsulation yield was 40.2±0.2%. No differences were observed in the %encapsulation yield amongst plasmolysed yeast cells when exposed to 10%, 20% or 30% w/w NaCl.

### Analytical studies for confirmation of berberine encapsulation

**Figure 4:**
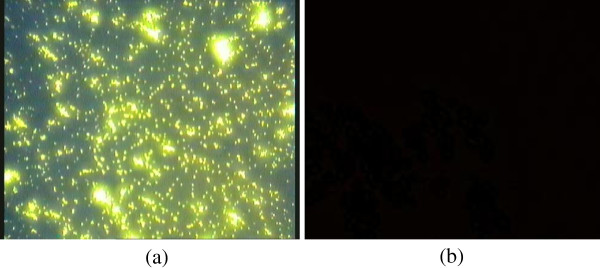
**Fluorescence micrographs of berberine yeast microcapsules (a) and empty *Saccharomyces cerevisiae *** **yeast cells (b).**

In the fluorescence micrographs, empty yeast cells could not emit fluorescence (Figure 4b). But the yeast cells loaded with berberine emitted fluorescence due to the presence of berberine which shows fluorescence properties (Figure 4a). This event proved the interaction of berberine with yeast cells. Further analyses were carried out using DSC and FT-IR to find out the effect of plasmolysis on cells structure.

Figure 1 showed the DSC thermograms of non-plasmolysed yeast cells, berberine, physical mixture of berberine and yeast cells as well as berberine microcapsules and plasmolysed yeast cells.

The new peak that appeared at 289°C which differed from the peak of empty yeast cells and physical mixture showed that the new product was produced. This Tm variation was due to decrease of Van der Waals interactions happened to membrane as a result of berberine’s integration. On the other hand, berberine influenced the bilayer organization by interaction of its polar groups with membrane polar groups. So the membrane became more fluid and Tm of microcapsules was lowered in comparison with empty yeast cells.

**Figure 5:**
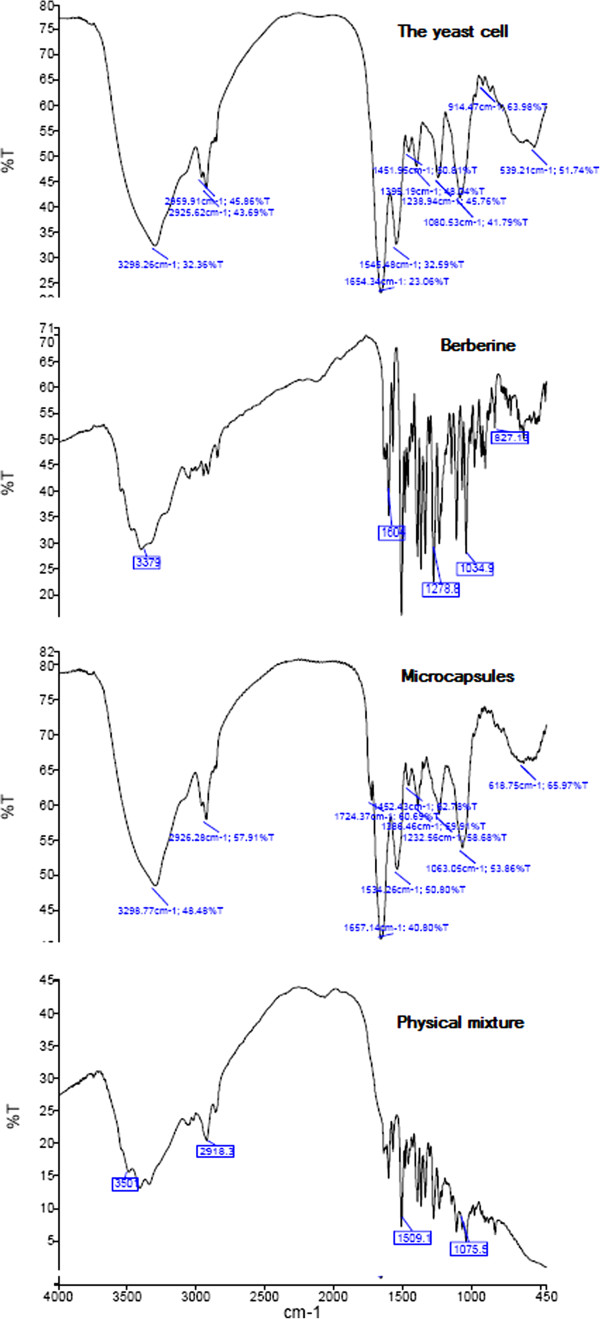
**FTIR spectra of *Saccharomyces cerevisiae *** **yeast cells, berberine, berberine microcapsules and physical mixture.**

The samples were studied with FTIR spectroscopy too and their spectra are showed in Figure 5. The interaction of berberine with yeast cell would alter the intensity of yeast cell absorption bands and causes shifts of characteristic bands.

The characteristic absorption bands of berberine are shown in Figure 5. The IR spectra of empty yeast cells are explained before. The 3298 and 1654 cm^-1^ bands are due to the phenolic and carbonyl groups, respectively. The absorption peak at 1545 cm^-1^ shows us the carbonyl and carbon-carbon vibration. The peak at 1451 cm^-1^ refered to carbon-hydrogen vibration. The absorption peak at 1238 cm^-1^ is indicative of phenolic vibration. The methoxy groups are responsible for the 1080 cm^-1^ band. The aromatic carbon-hydrogen group vibrates at 914 cm^-1^[34].

The IR spectrum of the physical mixture of berberine and yeast cells was a combination of the IR spectra of empty yeast cells and berberine, separately. The IR spectra of the microcapsules was relatively the same as the spectrum of the empty yeast cells. These spectra could prove the fact that berberine was placed inside the yeast cells. This observation confirmed Shi et al. [5,7] studies. However we had some peak disappearances at 914 cm^-1^ and variation in the absorption bands of 1654 and 1545 cm^-1^ as a result of berberine-protein interactions in microcapsules spectra.

## Conclusions

In this study, *S. cerevisiae* were introduced as a novel carrier for berberine as a model. It has shown that yeast microcapsules were loaded with berberine up to 40.2 ± 0.2% w/w. Three analytic methods indicated that berberine located inside the cells mainly by hydrophobic interactions with the yeast cell membrane and cell wall.

## Competing interests

The authors declare that they have no competing interests.

## Authors’ contribution

RS participated in project design, carried out analytic experiments and participated in drafting the manuscript. BS FB supervised the whole project, supported the culture of microorganism, and participated in drafting the manuscript. OR participated in project design and drafting the manuscript. ZK contribution in analytical methods, commented the analytical results and participated in drafting the manuscript. All authors read and approved the final manuscript.
